# Estimating the size of populations at risk for malaria: a case study in cattle herders and agricultural workers in Northern Namibia

**DOI:** 10.1038/s41598-024-56810-y

**Published:** 2024-03-26

**Authors:** Francois Rerolle, Jerry O. Jacobson, Cara Smith Gueye, Adam Bennett, Sidney Carrillo, Henry Ntuku, Jennifer L. Smith

**Affiliations:** Malaria Elimination Initiative, The Global Health Group, University of California, San Francisco, CA USA

**Keywords:** Malaria, Epidemiology

## Abstract

Cattle herders and agricultural workers have been identified has key high-risk populations for malaria in northern Namibia. Population size estimates for these groups are lacking but are important for planning, monitoring and evaluating the effectiveness of targeted strategies towards malaria elimination in the region. In this analysis, we extend population size estimation methods routinely used in HIV research, specifically social mapping and multiple source capture-recapture, to the context of malaria to estimate how many cattle herders and agricultural workers lived in two regions of northern Namibia over the course of the 2019–2020 malaria season. Both methods estimated two to three times more agricultural workers than cattle herders but size estimates based on the multiple source capture-recapture method were two to three times greater than the mapping-based, highlighting important methodological considerations to apply such methods to these highly mobile populations. In particular, we compared open versus closed populations assumptions for the capture-recapture method and assessed the impact of sensitivity analyses on the procedure to link records across multiple data sources on population size estimates. Our results are important for national control programs to target their resources and consider integrating routine population size estimation of high risk populations in their surveillance activities.

## Introduction

After years of steady decline, progress towards eliminating malaria has stalled across southern Africa and worldwide. Seasonal outbreaks of malaria in Namibia’s northern regions^1^ since 2016 have highlighted a need to identify coverage gaps and improve delivery of effective interventions^2^. Previous case–control studies and formative research conducted in two northern provinces, Ohangwena and Zambezi, identified specific occupations and behaviors that define malaria high-risk populations (HRP), as well as key intervention gaps^3,4^. Groups with high mobility and outdoor exposure to mosquitos, including seasonal agricultural workers (AW) and cattle herders (CH), are particularly challenging to access through routine surveillance and intervention strategies, which primarily target resident communities at their households. In low endemicity settings such as present-day northern Namibia, HRPs are thought to have a role in sustaining transmission and tailoring prevention and treatment efforts to address gaps in coverage is crucial in order to reach malaria elimination^5^. Yet, the population size of these groups is unknown and due to their high mobility, challenging to estimate through conventional methods.

An estimate of the population size of these groups is an essential input for planning, scaling, monitoring interventions and assess their coverage but also to understand and model patterns of transmission^6^. Population size estimates (PSE) can also mobilize resources and political will to support equitable malaria control programs^6^. When malaria risk is widespread in a community, size estimation can be as straightforward as conducting a household census, but when risk becomes more focused among individuals with specific occupations and behaviors or those who are harder-to-reach, more nuanced and targeted strategies are needed. This is particularly true in contexts where the activity leading to increased exposure may be informal, illicit or stigmatized.

Multiple studies^7–13^ and guidelines^6^ focusing on PSE for HRP in the context of HIV exist, but to our knowledge, there are no such equivalents for malaria. Yet, the most accepted PSE methods in use today in the HIV context originally hail from other disciplines and could be readily extended to other infectious diseases, such as malaria. For example, the capture-recapture method was developed in wildlife ecology to study population sizes of animals^14^, while the multiplier method and social mapping and enumeration have long been employed throughout the social sciences. When transmission is clustered among high-risk subpopulations and particularly hard-to-reach populations, researchers have recommended including PSE studies as a part of malaria surveillance systems^15^, following similar guidance for HIV surveillance^16,17^.

In this study, we illustrate adapting social mapping and multiple source capture-recapture to the context of malaria HRPs to estimate the population size of cattle herders and agricultural workers in Zambezi and Ohangwena Regions, in northern Namibia. Along the way, we highlight important methodological considerations to link ascertainments of individuals across multiple data sources and account for HRP’s mobility, and discuss challenges and opportunities for the routine use of these approaches.

## Methods

### Study context and overall estimation approach

**Figure 1:**
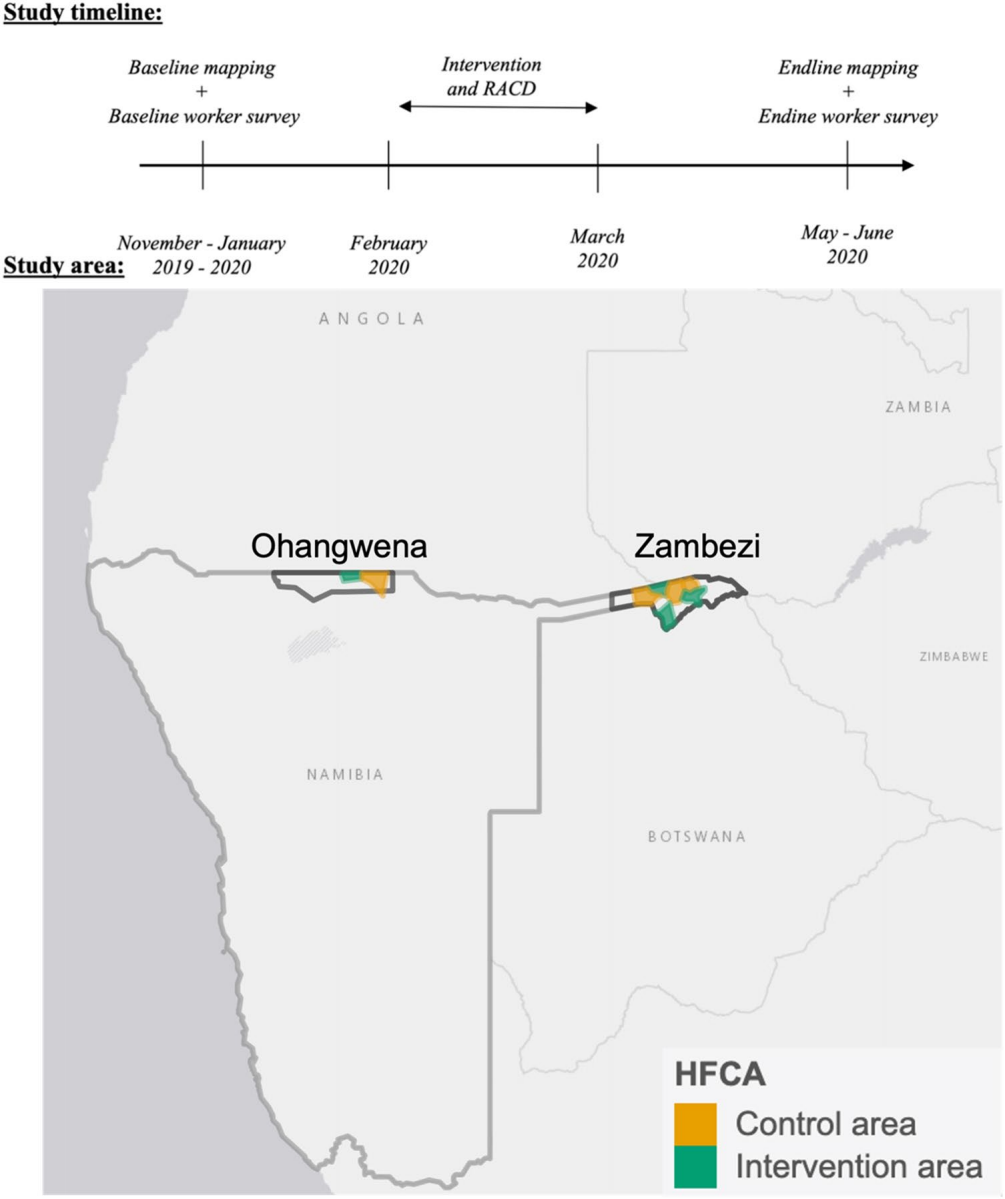
Study area and study timeline. Top: study timeline with intervention and RACD conducted between baseline and endline mapping and workers surveys. Bottom: study area in where 8 HFCAs randomly allocated to an intervention or control arm. ESRI imagery from the leaflet R packages was used for the basemap.

The PSE was planned as part of a quasi-experimental randomized controlled trial (NCT04094727; September 19, 2019) to evaluate the impact of a tailored package of interventions on malaria and coverage outcomes in agricultural workers and cattle herders in northern Namibia. The study was conducted over the 2019–2020 malaria season (November–June) in 8 health facility catchment areas (HFCA) across Zambezi and Ohangwena Regions, in northern Namibia (Fig. 1) where the total population was respectively 23,022 and 9995. The main trial comprised six components: (1) baseline mapping of worksites; (2) baseline worker survey; (3) delivery of malaria interventions in randomized HFCAs; (4) reactive case detection (RACD) at worksites; (5) endline mapping of worksites; (6) endline worker survey. These components differed for the two target populations and regions, as described below. Data collection to support size estimation was incorporated into each component from initial study conception. The mapping-based PSE drew on the baseline and endline mapping data; the multiple source capture-recapture PSE drew on the survey and intervention data. Table 1 lists the criteria to define high-risk agricultural workers and cattle herders in each survey.

**Table 1:** High-risk eligibility criteria for mapping, surveys, and interventions.

Data source	Zambezi	Ohangwena
Baseline and endline mapping of worksites	Cattle herders and/or agricultural workers employed by this owner who will sleep/slept or work(ed) outside at least one night at this farm/cattle post between Nov and May	Cattle herders employed by this owner who will spend/spent at least one night in Angola between Nov and May prior to returning to Ohangwena between Nov and May
Baseline and endline worker surveys	Primary occupation is agricultural worker or cattle herder Slept or worked outside at least one night at this farm/cattle post in past 1 week or will in upcoming 3 weeks	Primary occupation is cattle herder Spent at least one night working in Angola between Nov and May, in position as cattle herder
Interventions:		
Presumptive treatment	Agricultural worker or cattle herder workers who regularly sleep overnight at farms or cattle posts	Cattle herders who are cross-border travelers
IRS	Sprayable structures at farms or cattle posts that were not covered during the primary spray campaign	Sprayable structures at cattle posts located within 10 km of the border that were not covered during the primary spray campaign
Vector control pack	Those who do not sleep in a sprayed structure	Those who do not sleep in a sprayed structure in Angola
RACD	Agricultural worker or cattle herder workers who regularly sleep overnight at farms or cattle posts	Cattle herders who are cross-border travelers

### Data sources

#### Baseline and endline mapping of worksites

Mapping of worksites was conducted to generate a sampling frame for surveys and sites to target the intervention. Specifically, meetings were held with community health workers and community leaders in each region to develop a list of all farms and/or cattle posts that were thought to meet risk criteria. In Ohangwena, a veterinary services database was consulted to identify potential work sites; local leaders drew on this list and their knowledge of permits they had issued to cattle owners to authorize taking their cattle to and from Angola in order to limit the list to cattle posts where workers may engage in cross-border travel. Then, field teams conducted an interviewer-administered questionnaire with the owner or a manager at all worksites thus identified, with data collection by tablet. Data obtained included the number of workers expected to meet the high-risk population criteria defined in Table 1, over the course of the malaria season (i.e., November–May).

#### Baseline and endline workers surveys

The baseline and endline worker surveys were conducted among workers at a random sample of the worksites identified by the mapping. The surveys were interviewer-administered with data collection by tablet. The baseline and endline surveys were conducted at the beginning (November–January) and end (May–June) of the malaria season, respectively. Eligibility criteria were similar to those used in the mapping, however with a narrower time period of reference for the risk activity (see Table 1).

#### Intervention

The interventions were rolled out in four randomly sampled HFCAs between baseline and endline, during February and March. They included provision of presumptive treatment with artemether-lumefantrine to workers at worksites, indoor residual spraying (IRS) of worksite structures, and provision of a vector control pack to workers in Zambezi who did not sleep in a sprayed structure. Interventions were delivered in coordination with employers, at visits conducted independently of baseline, endline, and RACD surveys. Intervention participants were screened for eligibility (See Table 1). A second planned intervention round was interrupted by the SARS-CoV-2 pandemic in April 2020 and discontinued when Namibia entered lockdown.

#### RACD

The study team visited worksites to screen and interview co-workers of malaria cases reported by health facilities. See Table 1 for eligibility criteria. RACD was conducted in both intervention and control areas from February 2020 to March 2020, when it was discontinued due to the SARS-CoV-2 pandemic.

### PSE method 1: social mapping

The mapping-based size estimates were calculated in three steps based on worker counts obtained from worksites owners. First, the retrospective count reported by owners at endline were summed across worksites, based on the question item, “How many total workers [meeting the respective risk criteria] did you have from November 2019 to May 2020?”.

Second, to account for workers at worksites no longer operational by the endline mapping, we calculated a total prospective count reported during baseline interviews at these sites, based on a question item on the number of workers *expected* to meet the risk criteria between November 2019 and June 2020, which was otherwise identical to the endline item. Then, this sum was corrected for potential projection error by accounting for how the prospective and retrospective worker counts differed at sites that were included in both the baseline and endline. Specifically, the sum was multiplied by the ratio of the endline total divided by the baseline total. Next, this corrected count was added to the sum across endline sites calculated in the first step.

Finally, the result of the above was corrected for potential double-counting of workers who had worked at multiple sites in the respective region during the period by dividing by a mobility factor, which was calculated as the mean number of worksites per worker, based on responses to the endline survey question item, “How many employers/worksites in the [study region] have you worked for between November 2019 and today?”. The mobility adjustment factors and their associated 95% confidence intervals (CI) were estimated for each region and, in Zambezi, separately for agricultural workers and cattle herders. Dividing each summed count by the respective mobility point estimate and its 95% CI limits produced the corresponding PSE point estimate and its 95% CI.

### PSE method 2: multiple-source capture-recapture

The multiple source capture-recapture method draws on three or more statistically independent samples of the target population—all of which may be non-probability samples—and applies log-linear regression to estimate the population size based on patterns of overlap of individuals across these data sources or “captures”^18,19^. Here, four surveys—conducted at baseline, endline, during the intervention, and during RACD, respectively—served as the captures in each region.

#### Data management and record linkage

Birth place, birth order and three names variables (traditional, Christian and surname) were used to identify individuals across data sources. See appendix for details.

Records were linked using a flexible algorithm with a hierarchy of three different possible matching types:

- Perfect match: same 3 names in any order, same birth place and same birth order

or.

- Excellent match: same 3 names in any order and same birth place or same birth order

or.

- Good match: two of the same names in any order, same birth place and same birth order

Record linkage was first carried out within surveys for de-duplication and then across datasets to create capture histories for all records. When necessary, better matches were favored (e.g., perfect over excellent matches).

#### Sensitivity analysis to optimize record linkage parameters

We conducted sensitivity analysis to identify the optimal parameter settings for the clustering algorithms that were used to standardize the names and birth places reported by survey participants, as a preliminary step before record linkage. The two clustering algorithms in OpenRefine^20^ software were the n-gram fingerprint method (requiring a parameter *n*) and the Levenshtein nearest-neighbor method (requiring a radius of 1, 2, 3, or 4 and a block character setting of 3 or 4). These parameters determine the flexibility of the clustering; stricter settings may fail to identify similar spellings of names that in fact represent the same individual whereas overly flexible ones may erroneously cluster together the names of different respondents.

We selected the optimal values of the three parameters by repeating the clustering and record linkage procedure under all 24 possible combinations of the settings. Then, we manually reviewed a random sample of 100 records for which linkage results differed across the 24 parameter scenarios and classified the performance under each setting as correct or incorrect. Based on these samples, we calculated sensitivity and specificity and plotted a ROC curve to identify the parameter settings that produced the most accurate record linkage.

#### Statistical analysis

We developed capture histories (i.e., counts of individuals exhibiting each possible pattern of presence or absence across the four surveys) from the linked data. The capture histories were then analyzed by log-linear regression models^18^ using the RCapture^19^ package in R statistical software^21^ to produce the population size estimates. Models were developed under both closed- (i.e., no in- or out-migration) and open-population assumptions. The former allowed for heterogeneity in capture probabilities across surveys and across individuals.

Since the surveys and resulting model results reflect the intervention areas in each region, we applied an upweighting factor to extend the size estimates to control areas (where intervention surveys were not conducted) in the study area. Upweighting factors were calculated as the inverse of the proportion of baseline workers surveyed in intervention areas. Importantly, the baseline survey is assumed to be a representative sample of workers in the study areas and the relative proportion of workers in intervention versus control areas is assumed to be constant over the entire season.

### Ethics

This study was approved by Namibia’s Ministry of Health and Social Services (Approval #17/3/3HN), by the University of Namibia Research Ethics Committee (Approval #MRC/510/2019) and by the UCSF ethical review board (Approval #19-28530). The informed consent process was consistent with local norms, and all study areas had consultation meeting with, and approvals from, village elders. All participants provided informed written consent; caregivers provided consent for all children under 18. The study was conducted according to the ethical principles of the Declaration of Helsinki of October 2002.

## Results

### Social mapping PSE

In the Zambezi and Ohangwena regions respectively, 426 and 296 worksites were surveyed during both the baseline and endline mapping, with 1912 and 874 HRP workers projected to be employed over the season at baseline and 2353 and 731 HRPs reported to be employed over the season at endline. Therefore, projection factors applied to the prospective counts were 1.23 in Zambezi, where the sum total across sites at endline was greater than the total at baseline, and 0.84 in Ohangwena, where the situation was reversed.

Self reported levels of mobility of workers among worksites were modest. The mean number of worksites per worker from the endline survey was 1.07 [1.05; 1.08] for agricultural workers in Zambezi, 1.08 [1.05; 1.11] for cattle herders in Zambezi and 1.04 [1.02; 1.07] for cattle herders in Ohangwena. In Zambezi, 92.9% (N = 1121) of the 1207 endline survey participants reported working at one site and 7.1% (N = 86) had worked at two sites. In Ohangwena, 71.6% (N = 346) of 483 endline survey participants worked at one site, nine at two sites, and three at three sites, while 25.9% (N = 125) did not respond to the question item.

Table 2 shows the results from the mapping PSE. The calculation began with the number of workers reported in the endline mapping. Then, the number of workers projected in the baseline mapping worksites absent from the endline mapping, corrected by the projection factor, is added. Finally, the total is adjusted for mobility factors, yielding 724 [705; 745] cattle herders and 1914 [1896; 1950] agricultural workers in Zambezi and 725 [705; 739] cattle herders in Ohangwena.

**Table 2:** Mapping population size estimation.

	Zambezi Region		Ohangwena Region
Worksites			
Total	515		307
Worksites in endline mapping	432		300
Worksites both in endline and baseline mapping	426		296
Worksites in baseline mapping but absent from endline mapping	83		7
Workers	Cattle herders	Agricultural workers	Cattle herders
Total among endline sites	700	1656	738
Total among baseline sites not in endline, **prior to** applying correction factor	67	319	19
Total among baseline sites not in endline, **after** applying correction factor	82	392	16
Total among all sites, unadjusted for mobility	782	2048	754
Mobility adjustment factor [95% CI]	1.08 [1.05; 1.11]	1.07 [1.05; 1.08]	1.04 [1.02; 1.07]
Total among all sites, adjusted for mobility [95% CI]	724 [705; 745]	1914 [1896; 1950]	725 [705; 739]

### Multiple source capture-recapture PSE

#### Record linkage

Overall, response rates for the variables collected for purposes of record linkage (Table S1) were 90% or greater in the combined data across surveys and regions. Response rates were lower for Christian name in the Zambezi surveys (78–91%) and birth place and birth order in the RACD survey in Ohangwena (58%).

**Figure 2:**
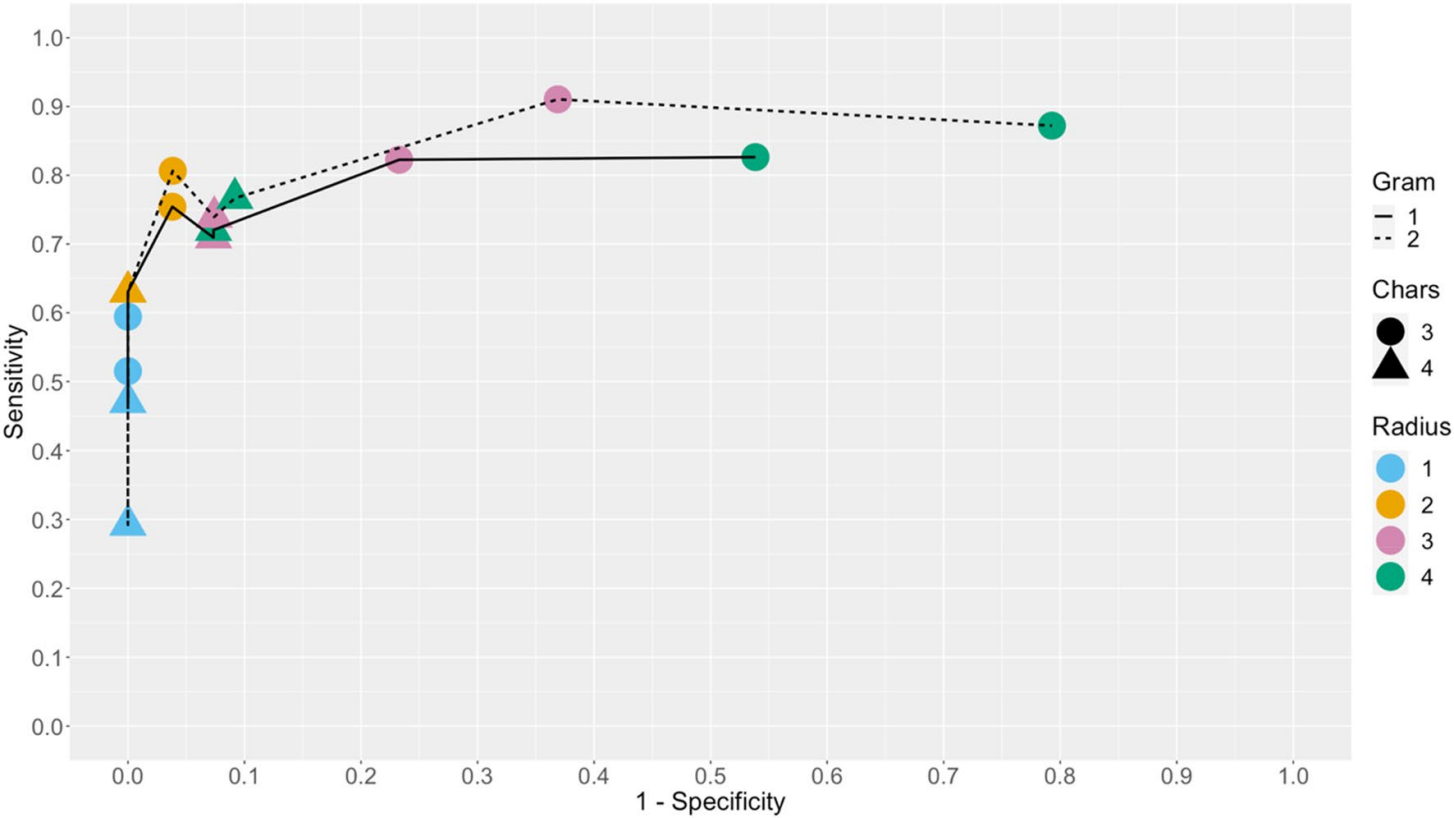
ROC curve. Assessment of how sensitivity and specificity change across the different standardization scenarios, with varying clustering parameters in OpenRefine: n in the n-gram fingerprint method; radius and block chars in the Levenshtein nearest-neighbor method. Scenarios with n-gram = 3 produced almost indistinguishable results as n-gram = 2 and were therefore discarded from the plot.

Varying standardization parameters in OpenRefine^20^ led to 24 scenarios that linked differently 651 (13%) of the total 5067 respondents across records. Among the review sample of 100 (15%), sensitivity and specificity varied quite a bit across scenarios (Fig. 2), ranging from 29 to 91% and 21 to 96% respectively. Two scenarios closest to the top left corner of the figure stand out. The first one (n-gram = 2, chars = 3, radius = 2, top left orange point) reached very good sensitivity (81%) while maintaining excellent specificity (96%) and was adopted in our subsequent PSE analyses. The second one (n-gram = 2, chars = 3, radius = 3, top centered pink point) had better sensitivity (91%) but at the expense of specificity (63%).

We also confirmed the extent to which combinations of the identifying variables for the adopted standardization parameters scenario uniquely identified records in the baseline and endline surveys, since to be eligible an individual should not have participated previously. Indeed, in our adopted record linkage algorithm, there were only 2 (0.2%) duplicated records in Zambezi’s baseline survey, none in Ohangwena’s baseline survey and 1 duplicated record in both Ohangwena and Zambezi’s endline surveys, representing 0.2% and 0.08% of records in the respective data source.

#### Statistical analysis

In the baseline survey in Ohangwena, 240 cattle herders were interviewed in the intervention areas and 194 in the control areas, yielding an upweighting factor of 1.8 (= [240 + 194]/240). In Zambezi, the upweighting factor was 2.2 (= [769 + 923]/769) for agricultural workers and 1.8 (= [505 + 404]/505) for cattle herders.

**Figure 3:**
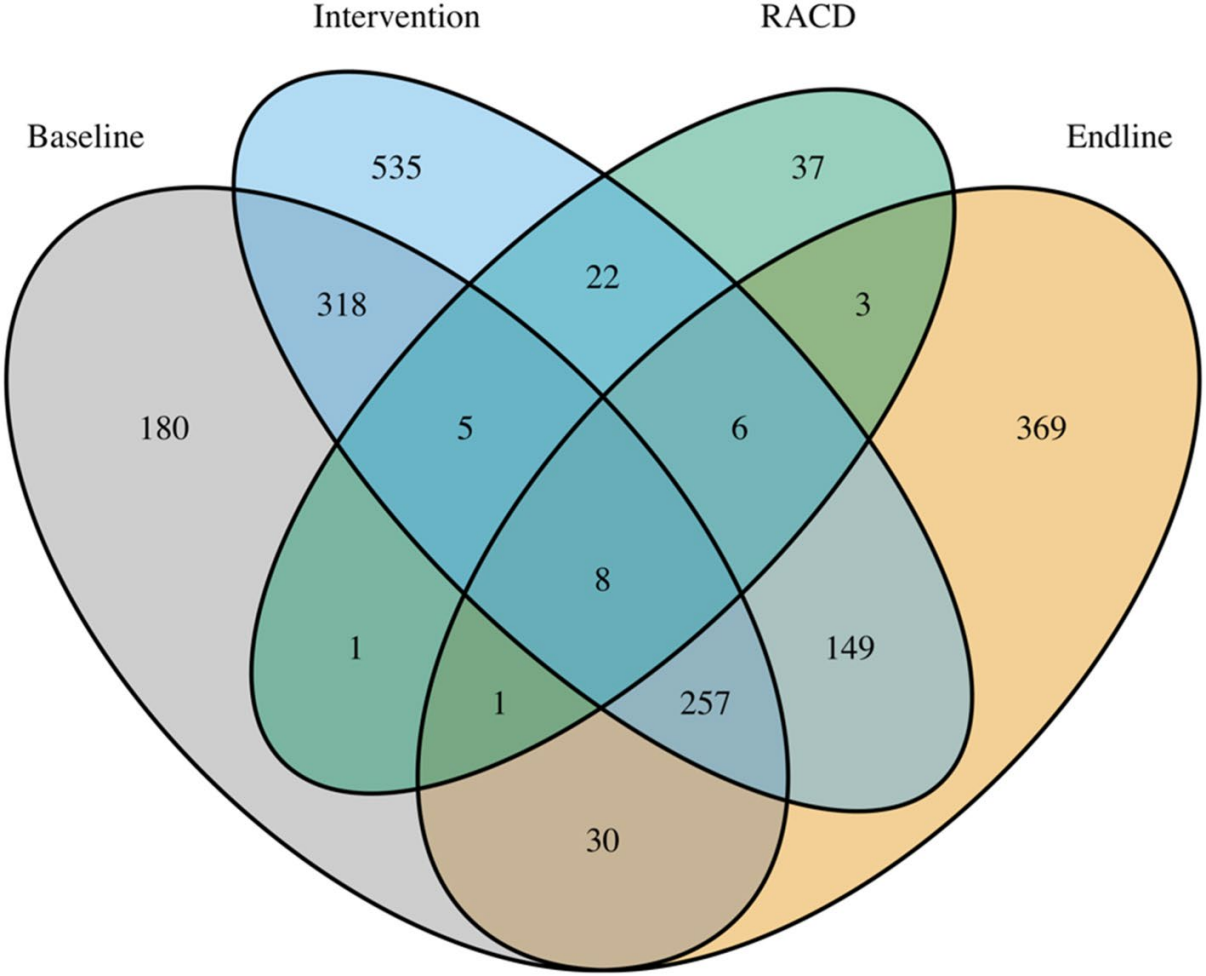
Venn diagram. Illustration of overlap of individuals across the four captures: for instance, 318 HRPs were captured both in the baseline and intervention surveys but not in RACD or endline surveys.

In the baseline, intervention, RACD and endline surveys in the intervention areas across both regions, a total of 800, 1300, 83, and 823 HRPs were ascertained, respectively, with 1921 unique HRPs based on record linkage. The Venn diagram in Fig. 3 illustrates the capture histories identified. See Figures S1, S2 and S3 in the appendix for similar Venn diagram stratified by region and high-risk groups.

Table 3 shows the results of the capture-recapture log-linear regression models, under closed- and open-population assumptions. Under the closed-population assumption, the best fitting model incorporated temporal and individual heterogeneity, allowing capture probabilities to vary both across surveys and workers (Mth models). Based on the AIC, the closed population model performed considerably better than open population models. In addition, the PSEs resulting from the open population models appear unsatisfactory as they are characterized either by an uninformative 95% CI (Ohangwena) or a point estimate only slightly above the total number of workers ascertained in surveys (upweighted to the study area). On the other hand, the open population model has the advantage of providing estimates of the baseline-intervention turnover rate, defined here as the probability a given worker leaves the area between baseline survey (November–January) and intervention (February–March). These turnover estimates were similar across population groups (13–20%) although non-significantly larger for cattle herders than agricultural workers in Zambezi.

**Table 3:** Capture-recapture PSE results.

		Ohangwena Cattle herders	Zambezi Cattle herders	Zambezi Agricultural workers
Total workers captured in intervention areas		594	404	923
Upweighting factor		1.8	1.8	2.2
Closed population	PSE [95% CI]	2225 [1706; 2745]	1908 [1211; 260*5*]	4316 [3472; 5159]
	AIC	85.5	75.1	98.7
	Model [Interaction terms]	Mth [BI, IR]	Mth [BI, BR]	Mth [BI, BR]
Open population	PSE [95% CI]	2552 [860; 4244]	931 [657; 1204]	2807 [2387; 3230]
	AIC	126.1	85.9	132.5
	Baseline-Intervention turnover [95% CI]	0.13 [0.05; 0.21]	0.2 [0.1; 0.29]	0.15 [0.09; 0.2]

Model estimates have been adjusted to include the upweighting factor. BI, IR and BR respectively stand for interaction terms between baseline/intervention, intervention/RACD and baseline/RACD data sources.

#### Sensitivity analysis of record linkage parameters

**Figure 4:**
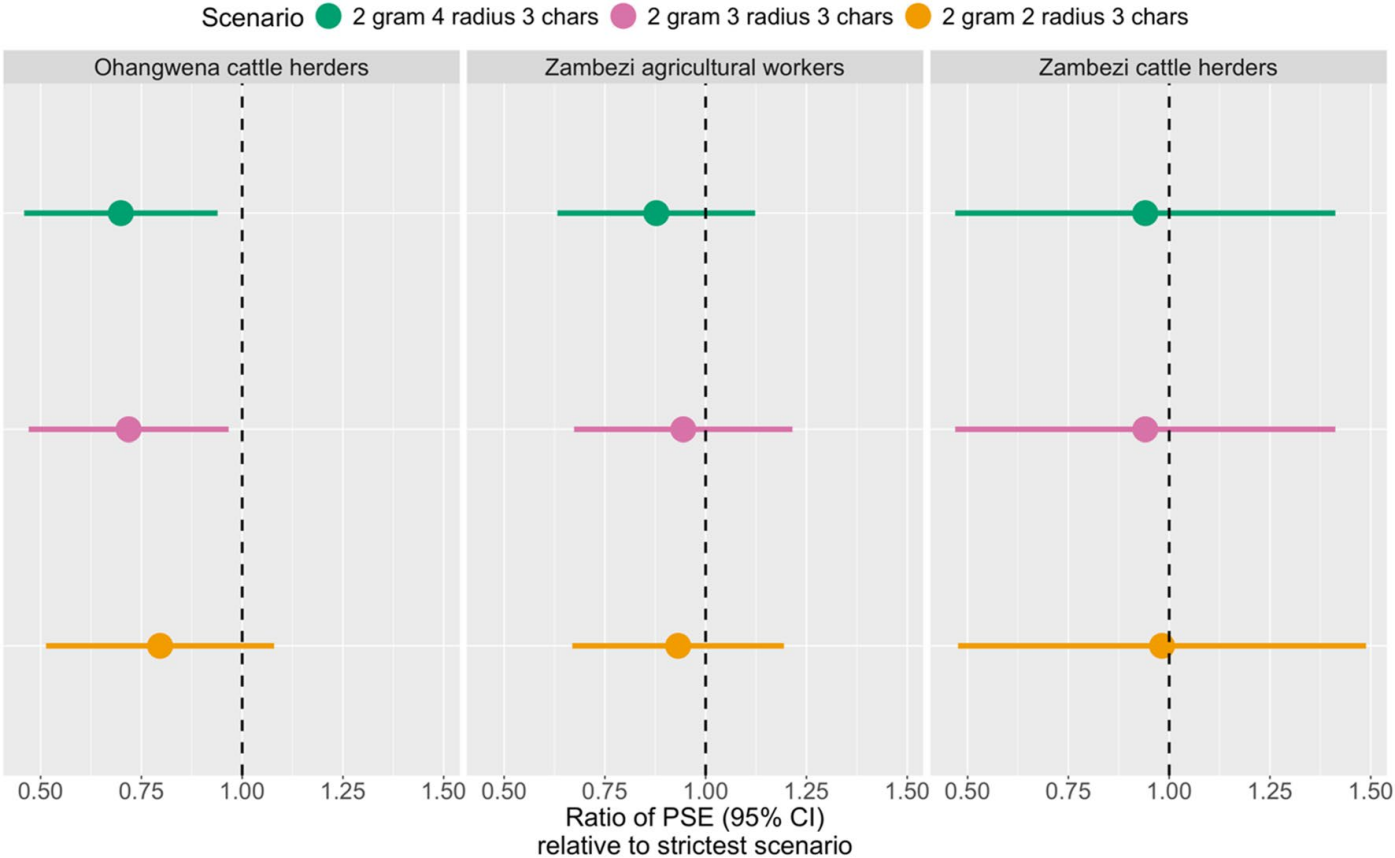
Impacts of standardization scenarios on PSEs. Assessment of how PSEs are impacted by standardization scenarios, with varying clustering parameters in OpenRefine: n in the n-gram fingerprint method; radius and block chars in the Levenshtein nearest-neighbor method. The dashed vertical black line represents the null where the PSE equals the PSE from the strictest scenario.

We ran the same closed population models on capture histories data resulting from the record linkage under four scenarios that vary parameterization of the clustering algorithm used to standardize names in the unique identifier: the strictest (n-gram = 1, radius = 1, block chars = 4), the most flexible (n-gram = 2, radius = 4, block chars = 3), the best (n-gram = 2, radius = 2, block chars = 3), and the second-best scenario (n-gram = 2, radius = 3, block chars = 3). These correspond respectively to the bottom left blue triangle, the top right pink circle, the top left orange circle, and the top center pink circle on Fig. 2. Figure 4 shows the ratio of the PSE obtained under each scenario relative to the strictest one (as the reference). The Delta method^22^ was used to compute 95% CI.

Greater flexibility in the record linkage algorithm yielded more matches across surveys, hence resulting in a smaller PSE (ie PSE ratio less than one). In Zambezi, none of the ratios are statistically different from one, meaning the standardization parameters scenarios would have resulted in non-significantly different PSEs. In Zambezi, the biggest difference occurred for agricultural workers with a PSE 0.88 [0.63; 1.12] times smaller in the most flexible scenario compared to the strictest one, representing an absolute difference of (2106–1848 =) 258 workers. In Ohangwena on the other hand, the most flexible and second-best scenarios yielded PSEs statistically significantly lower than the strictest ones with PSE ratios of 0.70 [0.46; 0.94] and 0.72 [0.47; 0.97] respectively. In Ohangwena, the best scenario resulted in a PSE 0.80 [0.51; 1.08] times smaller than the strictest scenario, but this was not statistically significant.

## Discussion

In this study, we leveraged two PSE methods to estimate the total population size, in our study area and over the 2019–2020 malaria season (November–June), of two occupational groups that met risk criteria derived from previous research in northern Namibia: cattle herders and agricultural workers. Size estimates based on the multiple source capture-recapture method (Table 3) were two to three times greater than the mapping-based estimates (Table 2). Similar differences are common in HIV size estimation studies, where mapping-based estimates are generally viewed as a lower bound^6^, highlighting the need to triangulate PSE results over several methods. Here, work site owners may have intentionally or unintentionally omitted employees whereas the capture-recapture method may have produced a more complete count by drawing on intervention and RACD data in addition to worksite surveys. Both methods estimated two to three times more agricultural workers than cattle herders in Zambezi suggesting that the former group may be more critical to malaria elimination; however, this also depends on the relative infection prevalence, which we did not assess here.

Our results point towards several methodological considerations of assumptions used in closed and open population models used in capture-recapture methods. While model fit and face validity of size estimates resulting from our closed population models appeared superior to those from our open population models, there is no gold standard ‘truth’ to empirically determine which is best. The epidemiological literature tends to focus on closed-population models^23^ because of short study time periods over which populations can assumed to be constant. Most prior literature on size estimates in high-risk populations derives from studies in the HIV context^6–12^ where surveys and other data captures are planned to span a brief time frame of about 3–5 months, precisely to limit the risk of change in the population over the course of the study. When aiming to ascertain the total risk population over the course of a several-month malaria season, such a strategy becomes infeasible for highly mobile populations and determining whether the population is truly open or closed over the period is a challenge^24^. The open-population models naturally accommodate the phenomenon of individuals entering and exiting the risk group over time, but even the so-called “closed” model can do so by introducing interaction terms that model temporal variation in the ascertainment probabilities of survey instruments, as was done here. When one has the data available (i.e., at least three data sources) to apply the log-linear^18,25,26^ regression model approach, comparing model fit under each scenario provides a way to assess the open versus closed assumptions.

A second methodological strength of this study that is new to PSE analyses was to vary record linkage scenarios, evaluate them and assess their impact on population size estimates. Capture-recapture methodology relies on the assumption that individuals can be tracked over different capture occasions. When it is not possible to collect a unique identifier, an object such as a study card^27^ can be given or, more commonly, a combination of identifying variables (names, gender, places of birth, places of residence, etc.…) are used to uniquely identify individuals across capture occasions. Yet, the selection of these variables, their standardization and the matching algorithms used can vary a lot and subjective decisions are often made on based on face validity. Here, identifying variables (names, birth place and birth order) as well as the matching algorithm (perfect, excellent and good matches) were selected subjectively but we varied standardization parameters to cover 24 different record-linkage scenarios. Comparing them highlighted quite some variability in terms of sensibility and specificity although, and importantly, any of these scenarios would have met face validity. This sensitivity analysis enabled us to choose an appropriate standardization scenario and assess how picking a scenario affects the population size estimates. Figure 4 showed that these scenarios would have not resulted in statistically significantly different estimates in Zambezi but would have led to different results in Ohangwena. These findings highlight the need for thorough assessment and transparent reporting of the quality of any record linkage algorithm used for population size estimates.

A first limitation of our analysis is that eligibility criteria across survey sources (Table 1) are not identical and may have ascertained different segments of the high-risk populations. In particular, eligibility criteria in the baseline and endline surveys pertain to narrow windows of time around the date of interview. Yet, these surveys were conducted over multiple weeks which means that, even within one survey, the criteria captured individuals from slightly different populations. Second, unique identifiers were based on self-reported variables which could lead to mismatches, further exacerbated by possible variations in how questions were elicited by different interviewers or answered by participants. To mitigate these limitations, we looked for and picked the best record linkage scenarios for our context, but sensitivity (81%) and specificity (96%) were not perfect. Because some matches were potentially missed, our estimates, if anything, could be viewed as upper boundaries of population sizes. Finally, the mapping exercise revealed some fluctuation in the number of worksites open between baseline and endline. In particular, the coronavirus crisis erupted in March 2020 and may have affected the overall population size for that particular year, limiting the transportability of our results to other more “normal” malaria seasons.

In conclusion, this study estimated the population size of high-risk populations for malaria in two regions of Northern Namibia. The significance of our work is threefold. First, the numerical population size estimates of key high-risk populations for malaria transmission in northern Namibia are important for national programs to target their resources and plan the delivery of their control interventions accordingly. Second, our study showcases how population size estimation methods can be leveraged in malaria research and discusses major methodological considerations for applying capture-recapture PSE to malaria’s high-risk populations. Last, our analysis used data routinely collected by national malaria control programs and proofreads the feasibility of integrating regular population size estimations into their surveillance activities.

### Supplementary Information

Supplementary Information 1.

Supplementary Information 2.

Supplementary Information 3.

## Data Availability

The datasets used in the multiplier method are available and published along the submission as supplementary materials. The datasets for the capture recapture method are not publicly available and cannot be de-identified since the record linkage algorithm relies on identifying variables (names, age, birth place). We still publish along the submission the datasets post-record-linkage (24 different scenarios form sensitivity analyses) and our for maximum transparency.
