# Supplementary material for: Estimating the size of populations at risk for malaria: a case study in cattle herders and agricultural workers in Northern Namibia
Source: Sci Rep. 2024 Mar 26;14:7160. doi: 10.1038/s41598-024-56810-y (PMC10965975; doi:10.1038/s41598-024-56810-y)
Supplement: Supplementary file 3 — Supplementary Information 3. [file 41598_2024_56810_MOESM3_ESM.docx]

**Supplementary materials for: Estimating the size of populations at risk for malaria: a case study in cattle herders and agricultural workers in Northern Namibia.**

Francois Rerolle^1*^, Jerry O. Jacobson^1^, Cara Smith Gueye^1^, Adam Bennett^1^, Sidney Carrillo^1^, Henry Ntuku^1^, Jennifer L. Smith^1^

^1^ Malaria Elimination Initiative, The Global Health Group, University of California, San Francisco, CA, USA

*For correspondence: rerollefrancois@gmail.com

- 1. **Supplementary tables**

| Survey | Baseline | | Endline | | Intervention | | RACD | |
| --- | --- | --- | --- | --- | --- | --- | --- | --- |
| Region | Ohangwena (N=434) | Zambezi  (N=1176) | Ohangwena (N=483) | Zambezi  (N=1207) | Ohangwena (N=503) | Zambezi  (N=987) | Ohangwena (N=57) | Zambezi  (N=220) |
| Birth order | 99.8 | 100 | 100 | 99.9 | 91.7 | 99.9 | 57.9 | 100 |
| Birth place | 99.8 | 99.9 | 99.8 | 99.9 | 96 | 100 | 57.9 | 100 |
| Traditional name | 97.2 | 99.6 | 98.8 | 98.3 | 95.8 | 99.8 | 100 | 97.7 |
| Christian name | 100 | 90.5 | 99.8 | 84.8 | 98.6 | 86.5 | 100 | 78.2 |
| Surname percent | 100 | 100 | 100 | 100 | 98.2 | 100 | 100 | 100 |

**Table S1 –** *Completeness (%) of identifying variables across surveys and regions.*

- 1. **Supplementary figures**


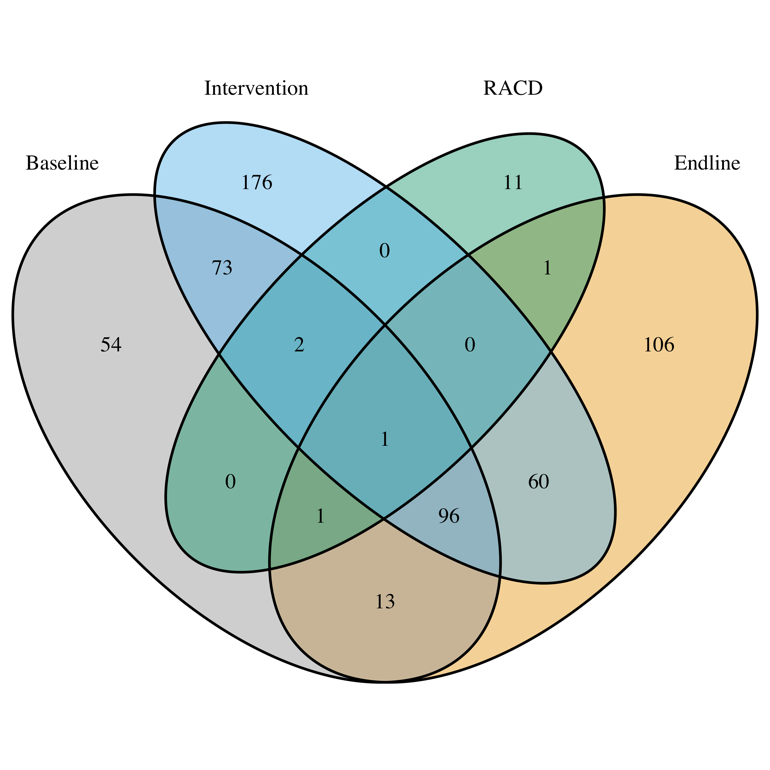


**Figure S1 – Venn diagram for cattle herders in Ohangwena.** *Illustration of the capture history data: for instance, 73 workers were captured both in the baseline and intervention surveys but not in RACD or endline surveys.*


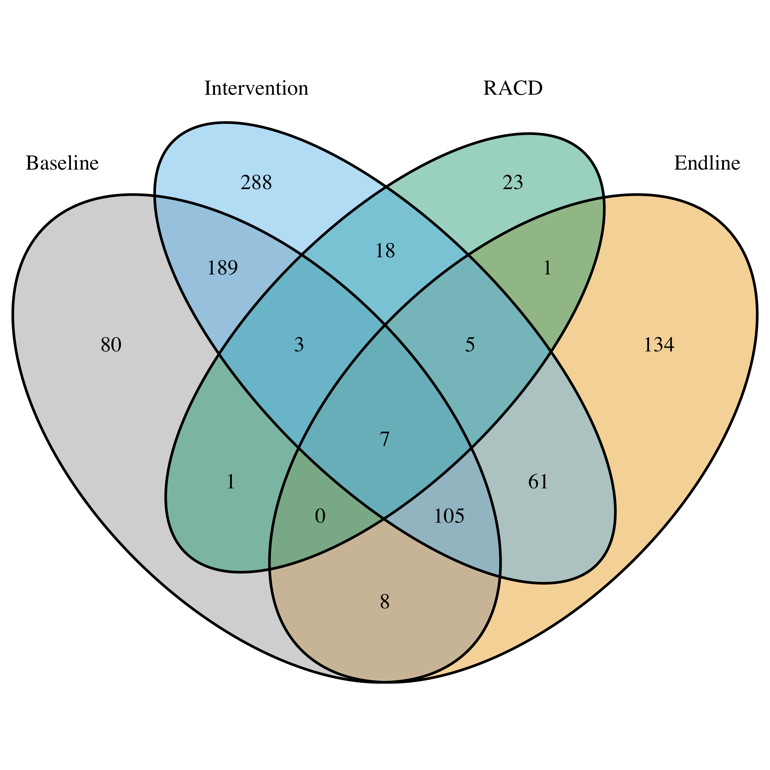


**Figure S2 – Venn diagram for agricultural workers in Zambezi.** *Illustration of the capture history data: for instance, 189 workers were captured both in the baseline and intervention surveys but not in RACD or endline surveys.*


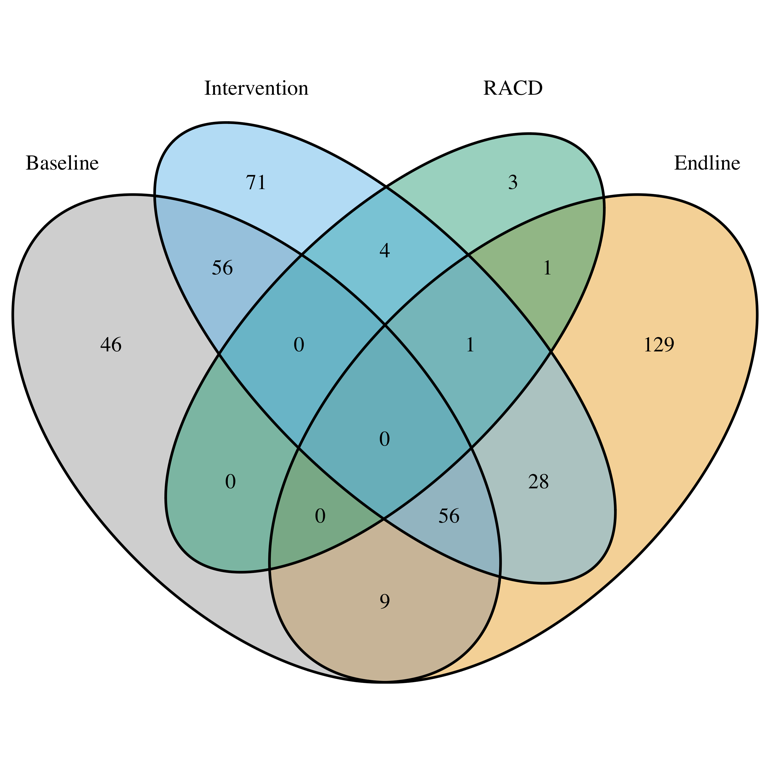


**Figure S3 – Venn diagram for cattle herders in Zambezi.** *Illustration of the capture history data: for instance, 56 workers were captured both in the baseline and intervention surveys but not in RACD or endline surveys.*

- 1. **Methodological details for PSE capture-recapture**
     1. **Standardization of names and birth places**

Initial review of the unique identifier data suggested variation in spelling of the three person names (traditional, Christian and surname) and birth place names across records for what otherwise appeared to be the same individuals. While interviewers were trained to request that participants spell out their names, we suspected that the variation might reflect differences in interviewers’ approaches to spelling, translation to the Latin alphabet, or unreliability of participants’ own spelling across interprets.

Standardization was conducted across datasets for both the three name variables together and birth place names. Names were converted to lower case and whitespaces were removed. Key collision, n-gram fingerprint and the Levenshtein nearest-neighbor matching algorithms were then applied with OpenRefine^20^ software to identify and standardize clusters of similar names. Birth place names were standardized further by deleting modifiers, including suffixes such as “village” and country names (Namibia, Angola, Zambia), since they did not appear to have been used consistently.

- - 1. **Record linkage**

Strict variable-by-variable linkage was not undertaken due to several factors. First, non-response on any variable would have prohibited linkage of otherwise matching records. Second, birth place was sometimes specified as a village or town (e.g., Shambolo) and sometimes more generally as a country or province (e.g., Zambia); such differences were present even across otherwise identical records. Moreover, traditional, Christian and surnames appeared to have been interchanged on otherwise identical records. There were also instances in which one record had a repeated name and the other a different name in its place (e.g., Johannes Johannes Nghifikwa vs. Bonya Johannes Nghifikwa, both born in Omboka and the 4th child on the maternal side).
